# Effects of Crude Extract of *Glycyrrhiza* Radix and *Atractylodes macrocephala* on Immune and Antioxidant Capacity of SPF White Leghorn Chickens in an Oxidative Stress Model

**DOI:** 10.3390/antiox13050578

**Published:** 2024-05-08

**Authors:** Chaosheng Zhang, Shaolong Wang, Yunsheng Han, Aijuan Zheng, Guohua Liu, Kun Meng, Peilong Yang, Zhimin Chen

**Affiliations:** Key Laboratory for Feed Biotechnology of the Ministry of Agriculture and Rural Affairs, Institute of Feed Research, Chinese Academy of Agriculture Sciences, Beijing 100081, China; 82101225429@caas.cn (C.Z.); 82101205294@caas.cn (S.W.); hanyunsheng@caas.cn (Y.H.); zhengaijuan@caas.cn (A.Z.); liuguohua@caas.cn (G.L.); mengkun@caas.cn (K.M.)

**Keywords:** crude extract of *Glycyrrhiza* radix and *Atractylodes macrocephala*, SPF white leghorn chicken, oxidative stress, anti-inflammation, antioxidant

## Abstract

The natural edible characteristics of Chinese herbs have led more and more people to study them as an alternative product to antibiotics. In this study, crude extracts of *Glycyrrhiza* radix and *Atractylodes macrocephala* (abbreviated as GRAM) with glycyrrhizic acid content not less than 0.2 mg/g were selected to evaluate the effects of GRAM on the immune and antioxidant capacity of model animals. Thirty 21-day-old male Leghorn chickens were weighed and randomly assigned to one of three groups of ten animals each. The treatments comprised a control group (CON), in which saline was injected at day 31, day 33, and day 35, an LPS-treated group (LPS), in which LPS (0.5 mg/kg of BW) was injected at day 31, day 33, and day 35, and finally a GRAM and LPS-treated group, (G-L) in which a GRAM-treated diet (at GRAM 2 g/kg) was fed from day 21 to day 35 with LPS injection (0.5 mg/kg of BW) at day 31, day 33, and day 35. The results of diarrhea grade and serum antioxidant measurement showed that the LPS group had obvious diarrhea symptoms, serum ROS and MDA were significantly increased, and T-AOC was significantly decreased. The oxidative stress model of LPS was successfully established. The results of immune and antioxidant indexes showed that feeding GRAM significantly decreased levels of the pro-inflammatory factors TNF-α, IL-1β, and IL-6 (*p* < 0.05) and significantly increased levels of the anti-inflammatory factors IL-4 and IL-10 and levels of the antioxidant enzymes GSH-Px and CAT (*p* < 0.05). GRAM resisted the influence of LPS on ileum morphology, liver, and immune organs and maintained normal index values for ileum morphology, liver, and immune organs. In summary, this study confirmed the antidiarrheal effect of GRAM, which improved the immune and antioxidant capacity of model animals by regulating inflammatory cytokine levels and antioxidant enzyme activity in poultry.

## 1. Introduction

Given the development of the trend of “green, efficient, and safe” in the feed industry and the stance of comprehensive “prohibition of antibiotics”, the antibiotic-replacement products we choose should not only improve the feed utilization rate and breeding efficiency but also meet the requirements of health, safety, and environmental protection [1,2]. So far, 117 kinds of natural plants with homologous characteristics of medicine and food have been included in China’s “Feed raw materials catalogue”, and studies have shown that some feed plants have antibacterial activity and immune-stimulating activity, as well as improving animal meat quality and reducing animal stress. Therefore, such natural forage plants with homologous characteristics of medicine and food are a good choice in the research and development of antibiotic-replacement products [3,4]. *Atractylodes macrocephala* is dried roots obtained from Asteraceae family of perennial herbaceous plants, which also includes *A. lancea* (Thunb.) DC and *A. chinensis* (DC.) Koidz. The plant have the capacity to relieve pain and stop diarrhea, dry out dampness and strengthen the spleen, dispel gas and the common cold, and tonify blood and vital energy in the context of traditional Chinese medical lore [5,6]. *A. macrocephala* is rich in active substances, such as ketone, β-eudesmol alcohol, and polysaccharides, which have good anti-inflammatory, antioxidant, bactericidal, and antiviral effects [7]. *Glycyrrhiza* radix is an ancient perennial leguminous herb in China. Its dry roots and rhizomes have the functions of replenishing vital energy and blood, regulating the spleen, removing heat toxicity in the body, alleviating excessive medicinal properties of other drugs, resolving phlegm and relieving cough, maintaining healthy digestive functioning of the stomach, and improving digestive and absorption ability [8,9]. *Glycyrrhiza* radix contains more than 20 kinds of triterpenoids and more than 300 kinds of flavonoids [10], among which the bioactive substances with anti-inflammatory and antioxidant properties mainly comprise flavones, coumarone, isoflavones, liquiritigenin, glycyrrhizin, and chalcone [11,12,13].

When oxidative stress occurs in livestock and poultry, it can damage the immune system and antioxidant system by affecting gene transcription, cell signal transmission, and enzyme and biological macromolecule activity [14,15], promote intestinal damage, immune suppression, and metabolic diseases, and seriously affect the animals’ growth and development [16]. *Glycyrrhiza* radix and *Atractylodes macrocephala*, which have antioxidant and anti-inflammatory qualities and a variety of biological functions [12,17], can be used as an ideal feed additive to improve immune and antioxidant capacity. Zhu L et al. [18] reported that glycyrrhizic acid can inhibit oxidative stress by activating the Keap1/Nrf2/HO-1 signaling pathway, thereby alleviating hypoxia-induced acute lung injury in fetal rats. Zhou Q et al. [19] reported that *Glycyrrhiza* interacted well with Keap1, promoted the transport of Nrf2 to the nucleus, and alleviated damage from H_2_O_2_ to A549 and Caco-2 cells. Tang T J et al. [20] reported that glycyrrhizin can promote AMPK phosphorylation and SIRT1 protein expression and inhibit the phosphorylation of nuclear factor NF-κB p65 to protect H9c2 cells from H_2_O_2_-induced cell damage. Li L et al. [21] reported that *Atractylodes* inhibited the expression of pro-inflammatory cytokines such as IL-6 and TNF-α and down-regulated the PI3K-AKT signaling pathway, thereby helping to enhance the repair of intestinal epithelial barrier function and alleviate colon damage in mouse colitis models. Shi K et al. [22] reported that *Atractylodes lancea* rhizome attenuates oxidative stress and inflammation through the TLR4/NF-κB and Keap1/Nrf2 signaling pathways, thereby alleviating LPS-induced acute lung injury. Above all, Glycyrrhiza radix and *Atractylodes macrocephala* contain active substances that can activate anti-inflammatory and antioxidant signaling pathways and improve the body’s immunity and the effect of antioxidant activity.

*Glycyrrhiza* radix and *Atractylodes macrocephala* have very good medicinal effects, but research on their anti-inflammatory and antioxidant capabilities in poultry farming is very scarce. Therefore, this study aimed to analyze *Atractylodes macrocephala* and *Glycyrrhiza* radix extract in an SPF white leghorn chicken model in terms of their effects on diarrhea and the immune system, as well as their antioxidant capacity, to explore the value of the application of GRAM in poultry nutrition as a substitute for antibiotics.

## 2. Materials and Methods

### 2.1. Preparing Materials

The 21-day-old SPF male White Leghorn chickens were bought from Beijing Boehringer Ingelhewiton Biotechnology Co., Ltd., Beijing, China. Lipopolysaccharide (LPS) of *E. coli* serotype O55:B5 was bought from Sigma-Aldrich Chemical Co., Shanghai, China. Buy GRAM from Baoding Jizhong Pharmaceutical Co., Ltd., Baoding, China.

### 2.2. Experimental Design and Bird Management

The animal test method was developed by the Feed Research Institute of the Chinese Academy of Agricultural Sciences, Beijing, China. This research report followed the ARRIVAL [23] guidelines and was finally implemented at the Nankou test base of the Chinese Academy of Agricultural Sciences, Nankou, China.

In the study, thirty healthy 21-day-old male SPF white leghorn chickens from the same batch that had been consistently fed and managed were randomly allocated into three groups with 10 chickens in each group. The treatments comprised a control group (CON), an LPS challenge group (LPS), and a GRAM intervention group (G-L). The control group (CON) and the LPS challenge group (LPS) were fed normally, while the GRAM intervention group (G-L) were fed a mixed diet containing 2 g/kg GRAM from day 21 to day 35. At 31, 33 and 35 days, the LPS challenge group (LPS) and the GRAM intervention group (G-L) were injected with 0.5 mg/kg body weight LPS intraperitoneally, while the control group (CON) was injected with normal saline intraperitoneally.

The illumination scheme was controlled in a cycle of 16 h illumination and 8 h darkness, the ambient temperature was maintained at 24 ± 2 °C until the end of the test, and the relative humidity was controlled at 50–60% from day 21 to day 35. Waste was cleaned out every day, and the air in the house was kept fresh. The experiment period lasted 14 days, and the chickens were fed a special diet for SPF chickens to meet their nutritional requirements. The nutritional formula of the diet is shown in Supplementary Table S1.

### 2.3. Diarrhea Index

On day 35 of the experiment, the fresh feces of the three groups of chickens were collected, weighed, recorded, and then dried in an oven at 65 °C to constant weight, which was the air-dried weight. Finally, the moisture content of chicken feces was calculated: fecal moisture content = (wet weight − air dry weight)/wet weight.

Diarrhea in each treatment group was observed on day 35, and each was scored according to Table 1. The higher the fecal water content and the higher the diarrhea grade, the more severe the diarrhea symptoms.

### 2.4. Sampling

On day 35, all chickens in the treatment group were fasted for 12 h, and then samples were collected. Blood samples were collected using 2.5 mL EDTA vacuum tubes and 5 mL anticoagulant-free vacuum tubes and immediately placed on ice. Blood samples from the anticoagulant-free vacuum tubes were centrifuged to collect serum and stored at −20 °C. After the blood sampling, the broilers were euthanized by electric stunning and immediate manual slaughter.

After the spleen, thymus, and bursa of Fabricius were removed, the fat and mesangium on their surfaces were carefully cut with surgical scissors, the blood on their surfaces was sucked with filter paper, and then weighed with electronic balance. The immune organ index was determined according to the production performance noun terms and metric statistics method for poultry.

The collection of ileum tissue was divided into two groups. Biochemical index analysis of ileum tissue samples from the first group was performed, which required the ileum segment to be put into a freezing tube for rapid freezing in liquid nitrogen and finally stored in a refrigerator at −80 °C. Morphological analysis of the ileal tissue from the other group was performed, which required fixation with 10% buffered neutral formaldehyde solution at pH 7.4.

### 2.5. Hematological and Serum Biochemical Index Analysis

The blood cell composition of anticoagulant blood collected in EDTA tubes was detected using a fully automated blood cell analyzer (Sysme XT-1800i, Shanghai, China). An Olympus AU640 automatic blood biochemical analyzer was used to detect the levels of alanine aminotransferase (ALT) and aspartate aminotransferase (AST). The preserved serum was collected using a commercial kit (Shanghai Enzyme-linked ImmunoBiotechnology Co., Ltd., Shanghai, China) and an enzyme labeler (MultiskanM™ SkyHigh, Thermo Fisher Scientific, Waltham, MA, USA) for antioxidant and immune markers. Levels of malondialdehyde (MDA), reactive oxygen species (ROS), glutathione peroxidase (GSH-Px), catalase (CAT), superoxide dismutase (SOD) and total antioxidant capacity (T-AOC) were used as serum antioxidant indexes. Levels of tumor necrosis factor (TNF-α), interleukin-1β (IL-1β), interleukin-4 (IL-4), interleukin-6 (IL-6), and interleukin-10 (IL-10) were taken as serum immune indicators.

### 2.6. Ileal Tissue-like Immune Index Analysis

The collected ileal tissue samples were prepared with a phosphate buffer solution to produce a 10% intestinal tissue homogenate at a 1:9 weight (g) to volume (mL) ratio, and then centrifuged with a high-speed refrigerated centrifuge at 4 °C, 4000 rpm, for 10 min to prepare the tissue homogenate supernatant. Inflammatory cytokines in the ileum were detected using a commercial kit from Enzyme Linked Biotechnology Co., Ltd., Shanghai, China. And an automated fluorescence instrument.

### 2.7. Histopathologic Analysis

All ileum tissues were dehydrated with anhydrous ethanol (75%, 85%, 95%, 100%), mixed with xylene (AR grade), embedded with paraffin wax, and made into 5 µm thick sections, then dewaxed and cleaned, and finally stained with conventional hematoxylin-eosin (H&E). A digital trinocular camera microscope (BA210Digital, MacOdy Industrial Group Co., Ltd., Singapore) acquired a magnified image of the intestinal segment ×40. Villus height and crypt depth were measured twice in the observed area (10 sets of data were measured each time), and the villus length/crypt depth ratio (V/C) was calculated.

### 2.8. Statistical Analysis

The data in this study were analyzed by one-way ANOVA using SPSS 19.0 Windows software (SPSS Inc., Chicago, IL, USA). Duncan’s multivariate range test was used to isolate significant differences among the groups. *p* < 0.05 was considered statistically significant.

## 3. Results

### 3.1. Establishment of Oxidative Stress Model of SPF Chicken Induced by LPS

In our study, diarrhea grade, fecal status, and T-AOC, MDA, and ROS levels were used as key indicators to determine the success of the LPS-induced oxidative stress model in SPF chickens. As shown in Table 2, compared with the CON group, diarrhea grade and fecal moisture content of chickens in the LPS group were significantly increased (*p* < 0.05), indicating that LPS stimulation caused obvious intestinal stress in chickens. At the same time, MDA and ROS in sera of chickens in the LPS group were significantly increased (*p* < 0.05) and T-AOC was significantly decreased (*p* < 0.05), indicating that the bodies of chickens stimulated by LPS produced oxidative stress responses. In conclusion, the oxidative stress model of SPF chickens induced by LPS was successfully established.

Based on the successful establishment of the SPF chicken model of oxidative stress induced by LPS, the effect of GRAM on oxidative stress response in the chickens was assessed (Table 2). Compared with the LPS group, diarrhea grade, fecal moisture content, and ROS and MDA levels in chickens in the G-L group were significantly decreased (*p* < 0.05). Compared with the CON group, fecal moisture and serum T-AOC in the G-L group were significantly decreased (*p* < 0.05), serum MDA was significantly increased (*p* < 0.05), but there was no significant difference in diarrhea grade or serum ROS (*p* > 0.05).

### 3.2. Effect of GRAM on the Histomorphology of Ileum in Chicken Model of Oxidative Stress

**Figure 1:**
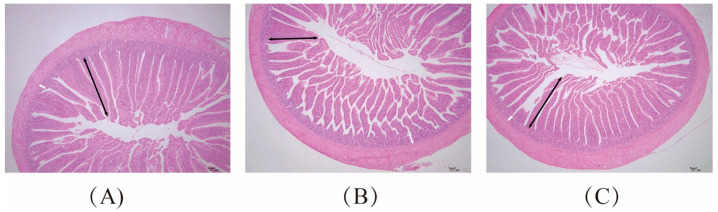
Section of ileum mucosa of chicken. Villus height (black arrow) and crypt depth (white arrow). (**A**) Control group; (**B**) LPS challenge group; (**C**) GRAM + LPS challenge group. Bar = 100 µm.

The effect of GRAM on the histomorphology of ilea in the chicken model of oxidative stress is shown in Table 3. Compared with the CON group, villus height and V/C ratio of ilea in the LPS group were significantly decreased (*p* < 0.05) and crypt depth was significantly increased (*p* < 0.05). Ileum mucosal tissue slices and measurement details of chickens in the control group are shown in Figure 1.

### 3.3. Effect of GRAM on Blood Routine Indexes in Chicken Model of Oxidative Stress

The effects of GRAM on blood routine indexes in the chicken model of oxidative stress are shown in Table 4. Compared with the CON group, LPS stimulation significantly increased blood WBC and GRA levels (*p* < 0.05) and significantly decreased blood LYM levels (*p* < 0.05), but there were no significant changes in blood HGB, RBC, or HCT levels (*p* > 0.05). Compared with the CON group, the levels of WBC, GRA, RBC and HCT in the G-L group were significantly increased (*p* < 0.05), the level of LYM was significantly decreased (*p* < 0.05), and the level of HGB was not significantly different (*p* > 0.05). Compared with the LPS group, RBC, HGB and HCT levels in the G-L group were significantly increased (*p* < 0.05), but WBC, GRA and LYM levels were not significantly different (*p* > 0.05).

### 3.4. Effect of GRAM on Liver Function Indexes in Chicken Model of Oxidative Stress

The effects of GRAM on the liver in the chicken model of oxidative stress are shown in Table 5. Compared with the CON group and the G-L group, LPS stimulation significantly increased serum ALT and AST levels and liver index values in chickens in the LPS group (*p* < 0.05). Compared with the CON group, the G-L group showed no significant difference in the three indexes (*p* > 0.05).

### 3.5. Effect of GRAM on Immune Organ Index in Chicken Model of Oxidative Stress

The effects of GRAM on immune organs in the chicken model of oxidative stress are shown in Table 6. The results showed that the spleen index and bursa of Fabricius index in the LPS group were significantly higher than those in the CON group and the G-L group (*p* < 0.05), while the spleen index in the G-L group was significantly higher than that in the CON group (*p* < 0.05). The thymus index showed no significant difference among the three treatment groups (*p* > 0.05).

### 3.6. Effect of GRAM on Serum Immune Index in Chicken Model of Oxidative Stress

The effects of GRAM on the serum cytokines in the chicken model of oxidative stress are shown in Table 7. Compared with the CON group, the levels of pro-inflammatory factors TNF-α, IL-1β and IL-6 in the LPS group were significantly increased (*p* < 0.05) and the levels of anti-inflammatory factors IL-4 and IL-10 were significantly decreased (*p* < 0.05), but there were no significant differences in cytokine indexes between the G-L group and the CON group (*p* > 0.05). Compared with the LPS group, the levels of pro-inflammatory factors TNF-α and IL-6 in the G-L group were significantly decreased (*p* < 0.05) and the levels of anti-inflammatory factors IL-4 and IL-10 were significantly increased (*p* < 0.05).

### 3.7. Effect of GRAM on Ileum Tissue Immune Index in Chicken Model of Oxidative Stress

The effects of GRAM on ileum tissue cytokines in the chicken model of oxidative stress are shown in Table 8. Compared with the CON group, the levels of pro-inflammatory factors TNF-α, IL-1β and IL-6 in the LPS group were significantly increased (*p* < 0.05) and the levels of anti-inflammatory factors IL-4 and IL-10 were significantly decreased (*p* < 0.05). However, only the pro-inflammatory factor IL-1β in the G-L group was significantly higher than that in the CON group (*p* < 0.05), and there was no statistical significance among the other indexes (*p* > 0.05). Compared with the LPS group, the levels of pro-inflammatory factors TNF-α and IL-6 in the G-L group were significantly decreased (*p* < 0.05) and the levels of anti-inflammatory factors IL-4 and IL-10 were significantly increased (*p* < 0.05).

### 3.8. Effect of GRAM on Serum Antioxidant Performance in Chicken Model of Oxidative Stress

The effects of GRAM on serum antioxidant performance in the chicken model of oxidative stress are shown in Table 9. Compared with the CON group, LPS stimulation significantly decreased GSH-Px, CAT, SOD, and NO levels in sera of chickens in the LPS group (*p* < 0.05). However, compared with the CON group, only serum SOD in the G-L group was significantly lower than the CON group (*p* < 0.05), and there was no significant difference among other indexes (*p* > 0.05). Compared with the LPS group, serum GSH-Px, CAT, and NO levels in the G-L group were significantly increased (*p* < 0.05).

## 4. Discussion

Lipopolysaccharide (LPS) is a component of the outer wall of Gram-negative bacterial cells and is mainly composed of lipids and polysaccharides, which can damage the immune system and intestinal structure of poultry and lead to a decline in poultry production performance [24,25,26]. Bosmann, M et al. [27] found that excessive endogenous factors released by LPS are the main substances causing oxidative stress and inflammation in the body, and the LPS-induced oxidative stress model has practical significance in many studies [28,29]. In this study, animals were injected with LPS to simulate bacterial infection. LPS-induced intestinal injury is accompanied by the destruction of antioxidant homeostasis [30]. Serum T-AOC, ROS, and MDA are important indicators for evaluating antioxidant activity, so this study selected them together with fecal indicators as the criteria for establishing a successful oxidative stress model. In our study, the oxidative stress model of SPF chickens showed that watery feces appeared after LPS stimulation, and the diarrhea grade, fecal moisture content, and serum ROS and MDA of model chickens were significantly increased, while serum T-AOC was significantly decreased. Therefore, we concluded that the SPF chicken model of oxidative stress induced by LPS was successfully established. On this basis, after feeding GRAM to model chickens, it was found that the diarrhea grade, fecal moisture content, and serum ROS and MDA of chickens were significantly reduced, indicating that GRAM inhibited the oxidative stress response in model chickens and alleviated their diarrhea symptoms. Whether GRAM can be as effective in real infections and whether other herbs have similar effects will require more trials.

The increase in villus height, the decrease in crypt depth, and the increase in villus height-to-crypt depth ratio are all features of the improvement in intestinal tissue morphology and structure, and are conducive to the digestion and absorption of nutrients and improved antistress and intestinal barrier functions of livestock and poultry [31]. The ileum histological results of chickens in this study showed that compared with the CON group, the villus height and V/C ratio of the ileum of chickens in the LPS group were significantly reduced, while the crypt depth was significantly increased, so LPS did induce structural damage in the ileum of chickens. However, after feeding GRAM to model chickens, compared with the LPS group, the villus height, crypt depth and V/C ratio of ileum in the G-L group were significantly increased and were maintained at the same level as those in the CON group. Therefore, glycyrrhiza radix and Atractylodes macrocephala can through the intestinal tissue repair damage and increase the intestinal absorption area, thus increasing the capacity of livestock and poultry to digest and absorb nutrients. Wen X B et al. [32] reported that intraperitoneal injection of LPS reduced the villus height in the ilea of piglets, resulting in intestinal damage. You T et al. [33] reported that dietary supplementation with licorice flavone powder significantly increased duodenal villus height and V/C ratio and decreased duodenal crypt depth in piglets. Liu B H et al. [34] also reported that feeding Chinese herbal feed additives mainly containing Atractylodes significantly reduced the crypt depth of the ileum and increase the density of jejunum cup cells of laying hens, thus improving the intestinal health of hens. These conclusions are consistent with the results of this study, and some studies have pointed out that enhancing the activity of antioxidant enzymes can alleviate LPS-induced apoptosis and reduce intestinal mucosal damage [35,36]. Therefore, the effect of GRAM on alleviating intestinal damage may be due to its enhancement of the activity of antioxidant enzymes in model chickens.

Each component in the blood is the material basis of metabolism, and changes in content can reflect changes in metabolic function, the metabolism of nutrients, and the function of tissues and organs of the body, and ultimately reflect the health status or disease status of livestock and poultry [37]. Leukocytes are important immune cells and an important index to reflect the level of immune response [38]. The results of chicken blood routine indexes in this study showed that LPS stimulation significantly increased the levels of WBC and GRA in the blood of model chickens and significantly decreased the levels of LYM, indicating that LPS, a Gram-positive bacterial antigen, caused bacterial infection and activated the humoral immune system of chickens. Liu J et al. [39] reported that blood WBC and LYM levels of laying hens were significantly increased after intraperitoneal injection of LPS. However, after feeding GRAM to model chickens, RBC, HGB, and HCT levels in blood increased significantly, while WBC and GRA levels decreased without statistical significance. These shows that glycyrrhiza radix and Atractylodes macrocephala enhance the body’s ability to transport oxygen and a large number of red blood cells and hemoglobin are combined to transport to various tissues and organs, thereby improving the body’s metabolic capacity, but with no significant effect on the body’s immune performance. Toson E et al. [40] also reported that supplementation with glycyrrhiza extract in broiler diets increased blood WBC, RBC and HGB levels.

The liver is the main site of amino acid metabolism. When liver cells are injured or the liver is affected by inflammation, the body’s protein metabolism is slow and ALT and AST are released, resulting in increased serum ATL and AST levels [41]. In this study, serum ATL and AST and liver index values were significantly increased after intraperitoneal injection of LPS, indicating that the liver was damaged and liver function was impaired after acute infection. Mireles et al. [42] reported that the injection of LPS in broilers induced infection and increased liver weight. After feeding GRAM to model chickens, the levels of ALT, AST and liver index values in serum were significantly decreased, and all remained at the same level as the CON group, indicating that GRAM alleviated liver damage induced by LPS in chickens. Gou S H et al. [43] reported that total flavonoids from glycyrrhiza significantly reduced serum AST and ATL in mouse models with liver injury and had a certain hepatoprotective effect. Miao Y F et al. [44] also reported that feeding chickens a certain dose of Atractylodes polysaccharide can significantly reduce serum AST and ATL, which can protect against liver injury. These reports are consistent with the results of this study, confirming the efficacy of GRAM in alleviating liver damage.

The strength of immune function is closely related to the developmental state of immune organs. The thymus, bursa of Fabricius, and spleen are important immune organs in poultry, and their organ index values can be used to evaluate the developmental state of immune organs of the body [45]. In this study, the spleen index and bursa of Fabricius index were significantly increased after intraperitoneal injection of LPS, indicating that LPS stimulated and activated the immune system and induced the proliferation and development of immune organs. Liu S D et al. [46] and Li Y et al. [47] reported that the injection of LPS in broilers would cause immune stress, thus stimulating the abnormal growth of immune organs, resulting in an abnormal increase in spleen index and bursa of Fabricius index values. Tang L P et al. [48] reported that heat stress increased spleen index and bursa index values in broilers, which was due to the edema of immune organs. However, the spleen index value of the model chickens was significantly reduced after feeding GRAM, and the bursa index was maintained at the same level as that of the CON group. These results indicated that GRAM can inhibit the abnormal proliferation of immune organs induced by LPS and maintain the normal development of immune organs in broilers.

When the body is injured, the inflammatory cells in the body will be activated, resulting in the release of inflammatory cytokines, and the level of inflammatory cytokines in the body will increase [49]. The results of this study showed that intraperitoneal injection of LPS significantly increased the levels of pro-inflammatory factors TNF-α, IL-1β, and IL-6 in sera and ilea and significantly decreased the levels of anti-inflammatory factors IL-4 and IL-10. However, after feeding GRAM to model chickens, the serum levels of pro-inflammatory factors TNF-α and IL-6 were significantly decreased, and the levels of pro-inflammatory factor TNF-α in ilea were also significantly decreased, while the levels of anti-inflammatory factors IL-4 and IL-10 in sera and intestinal tissues were significantly increased. This indicates that GRAM can induce the secretion of anti-inflammatory factors and inhibit the intestinal inflammatory response induced by LPS. Li W et al. [50] reported that LPS induced increased the expression of IL-1β and IL-6 in the spleen of geese; however, dietary supplementation of Atractylodes polysaccharide decreased the levels of oxidative stress and inflammatory cytokines induced by LPS. Wu Y et al. [51] reported similar results: glycyrrhiza polysaccharides increase the intestinal anti-inflammatory factors IL-4 and IFN-γ in chickens, thus improving cellular immune response. You T et al. [33] and Yin S et al. [52] reported that dietary supplementation with licorice flavone powder decreased IL-1β levels and increased IL-10 levels in duodena and spleens of weaned piglets.

GSH-Px, CAT and SOD are the main antioxidant enzymes of the body’s enzyme-promoted antioxidant system, and their activity and levels can be used as indexes to evaluate the body’s antioxidant status. Li L et al. [53] reported that the down-regulation of GSH-PX expression was related to kidney injury in the body, and serum creatinine level was an important biochemical index for evaluating kidney injury [54]. Wang, M et al. [55] also reported that GSH-PX expression decreased when kidney injury occurred in the body and increased when the kidney injury was relieved. In this study, GSH-Px, CAT, SOD, and NO levels in sera of model chickens were significantly reduced after LPS stimulation, indicating that the kidney may be damaged at the same time as oxidative stress in the body. However, after the chickens were fed GRAM, serum GSH-Px, CAT, and NO levels of the model chickens were significantly increased and maintained at the same level as that of the CON group, indicating that GRAM alleviates the LPS-induced oxidative stress response of the body and may also alleviate the kidney injury in the model chickens by regulating the expression of antioxidant enzymes in vivo. Li W et al. [50] reported similar results: dietary supplementation with Atractylodes polysaccharide alleviated LPS-induced oxidative stress, and the concentrations of MDA, T-AOC and GSH in spleens of geese returned to the normal range. Yin S et al. [52] reported that dietary supplementation with glycyrrhiza flavonoid powder increased the levels of T-AOC, GSH-Px, and SOD in sera of piglets and improve antioxidant activity in the body. GSH-Px and SOD are typical phase II metabolic enzymes, so feeding GRAM may improve the intestinal antioxidant capacity of chickens by regulating the expression of intestinal antioxidant enzymes and related genes, and ultimately improve the ability of the whole body to resist oxidative stress. The efficacy of GRAM in alleviating kidney injury requires serum creatinine and histological analysis in follow-up studies.

## 5. Conclusions

To sum up, an oxidative stress model of SPF chickens was successfully established using LPS, which led to intestinal inflammation and oxidative stress responses. Adding GRAM to the diet of chickens can alleviate the symptoms of diarrhea and oxidative stress induced by LPS and increase the levels of anti-inflammatory factors IL-4 and IL-10 and antioxidant enzymes GSH-Px and CAT. The levels of TNF-α, IL-1β, IL-6 and ROS and MDA were decreased, and the immune and antioxidant properties of model chickens were improved. The results of this study also showed that GRAM can resist the invasion and damage from LPS to the ileum, liver, and immune organs and maintain the normal development thereof. GRAM, a natural plant with homologous characteristics of medicine and food, has great potential in the research field of “antibiotic substitute” feed additives for poultry, and the relationship between its antidiarrheal effect and anti-inflammatory and antioxidant properties is worthy of further study.

## Figures and Tables

**Table 1:** Grading rules for diarrhea.

Grade of Diarrhea	Fecal Condition
0	Normal stool
1	Mild diarrhea with wet and soft stools
2	Moderate diarrhea with loose stools and perianal staining
3	Severe diarrhea with watery stools and severe perianal staining occurred

**Table 2:** Effect of GRAM on diarrhea grade, fecal status, and serum antioxidant levels (*N* = 10).

Items	Experimental Treatment			SEM	*p*-Value
	CON	LPS	G-L		
Diarrhea grade	1.33 ^a^	3.33 ^b^	1.17 ^a^	0.262	<0.001
Fecal moisture content	19.66 ^b^	26.73 ^a^	18.45 ^c^	1.292	<0.001
T-AOC, mmol Trolox/L	0.10 ^b^	0.06 ^a^	0.07 ^a^	0.005	<0.001
MDA, nmol/mL	6.93 ^a^	9.09 ^c^	7.95 ^b^	0.288	<0.001
ROS	210.58 ^a^	266.90 ^b^	215.00 ^a^	9.410	0.005

CON = control group; LPS = LPS challenge group; G-L = GRAM + LPS challenge group; T-AOC, total antioxidant capacity; MDA, malondialdehyde; ROS, reactive oxygen species. Different lowercase letters in the same line indicate significant differences (*p* < 0.05). SEM = standard error of means.

**Table 3:** The effect of GRAM on the ileum morphology of chickens (*N* = 10).

Items	Experimental Treatment			SEM	*p*-Value
	CON	LPS	G-L		
Villus height, μm	770.80 ^b^	624.06 ^a^	666.13 ^ab^	25.330	0.038
Crypt depth, μm	116.66 ^a^	145.80 ^b^	105.39 ^a^	6.271	0.005
V/C ratio	6.78 ^b^	4.32 ^a^	6.34 ^b^	0.386	0.003

CON = control group; LPS = LPS challenge group; G-L = GRAM + LPS challenge group. V/C = villus height (μm)/crypt depth (μm). Different lowercase letters in the same line indicate significant differences (*p* < 0.05). SEM = standard error of means.

**Table 4:** The effect of GRAM on the blood routine test of chickens (*N* = 10).

Items	Experimental Treatment			SEM	*p*-Value
	CON	LPS	G-L		
WBC, 109/L	104.43 ^a^	116.30 ^b^	112.96 ^b^	1.952	0.018
GRA, 109/L	29.15 ^a^	44.60 ^b^	39.80 ^b^	2.363	0.005
LYM, 109/L	60.70 ^b^	57.04 ^a^	57.52 ^a^	0.587	0.006
HGB, g/L	127.33 ^ab^	119.00 ^a^	138.33 ^b^	2.812	0.008
RBC, 1010/L	220.33 ^a^	220.63 ^a^	243.33 ^b^	3.443	0.010
HCT, L/L	0.21 ^a^	0.20 ^a^	0.23 ^b^	0.004	0.006

CON = control group; LPS = LPS challenge group; G-L = GRAM + LPS challenge group. WBC, white blood cell count; GRA, granulocyte count; LYM, lymphocyte; HGB, hemoglobin concentration; RBC, red blood cell count; HCT, hematocrit. Different lowercase letters in the same line indicate significant differences (*p* < 0.05). SEM = standard error of means.

**Table 5:** The effect of GRAM on the liver of chickens (*N* = 10).

Items	Experimental Treatment			SEM	*p*-Value
	CON	LPS	G-L		
ALT, U/L	61.80 ^a^	77.02 ^b^	67.68 ^a^	2.147	0.001
AST, U/L	264.22 ^a^	394.97 ^b^	317.19 ^a^	16.512	<0.001
Liver index, %	2.28 ^a^	2.69 ^b^	2.32 ^a^	0.073	0.016

CON = control group; LPS = LPS challenge group; G-L = GRAM + LPS challenge group; ALT, alanine aminotransferase; AST, aspartate aminotransferase. Different lowercase letters in the same line indicate significant differences (*p* < 0.05). SEM = standard error of means.

**Table 6:** The effects of GRAM on the immune organs of chickens (*N* = 10).

Items	Experimental Treatment			SEM	*p*-Value
	CON	LPS	G-L		
Spleen index, %	0.20 ^a^	0.38 ^c^	0.31 ^b^	0.023	<0.001
Thymus index, %	0.82	0.90	0.86	0.035	0.657
Bursa of Fabricius index, %	0.48 ^a^	0.72 ^b^	0.44 ^a^	0.040	<0.001

CON = control group; LPS = LPS challenge group; G-L = GRAM + LPS challenge group. Different lowercase letters in the same line indicate significant differences (*p* < 0.05). SEM = standard error of means.

**Table 7:** The effects of GRAM on the serum cytokines of chickens (*N* = 10).

Items	Experimental Treatment			SEM	*p*-Value
	CON	LPS	G-L		
TNF-α, pg/mL	40.13 ^a^	53.94 ^b^	44.43 ^a^	1.968	0.001
IL-1β, pg/mL	405.93 ^a^	520.44 ^b^	461.57 ^ab^	18.821	0.013
IL-6, pg/mL	14.53 ^a^	20.77 ^b^	14.55 ^a^	0.888	<0.001
IL-4, pg/mL	125.84 ^b^	86.01 ^a^	109.94 ^b^	6.170	0.005
IL-10, pg/mL	68.73 ^b^	48.55 ^a^	63.53 ^b^	2.544	<0.001

CON = control group; LPS = LPS challenge group; G-L = GRAM + LPS challenge group; TNF-α, tumor necrosis factor; IL-1β, interleukin-1β; IL-6, interleukin-6; IL-4, interleukin-4; IL-10, interleukin-10. Different lowercase letters in the same line indicate significant differences (*p* < 0.05). SEM = standard error of means.

**Table 8:** The effects of GRAM on ileum tissue cytokines of chickens (*N* = 10).

Items	Experimental Treatment			SEM	*p*-Value
	CON	LPS	G-L		
TNF-α, pg/mL	34.53 ^a^	50.60 ^b^	41.72 ^a^	2.281	0.001
IL-1β, pg/mL	348.07 ^a^	494.21 ^c^	419.21 ^b^	19.100	<0.001
IL-6, pg/mL	13.14 ^a^	16.36 ^b^	14.28 ^ab^	0.597	0.036
IL-4, pg/mL	113.54 ^b^	80.57 ^a^	103.11 ^b^	4.649	0.001
IL-10, pg/mL	61.53 ^a^	39.23 ^b^	55.82 ^a^	2.731	<0.001

CON = control group; LPS = LPS challenge group; G-L = GRAM + LPS challenge group; TNF-α, tumor necrosis factor; IL-1β, interleukin-1β; IL-6, interleukin-6; IL-4, interleukin-4; IL-10, interleukin-10. Different lowercase letters in the same line indicate significant differences (*p* < 0.05). SEM = standard error of means.

**Table 9:** The effects of GRAM on the serum antioxidant performance of chickens (*N* = 10).

Items	Experimental Treatment			SEM	*p*-Value
	CON	LPS	G-L		
GSH-Px, U/mL	124.28 ^b^	87.29 ^a^	112.04 ^b^	4.913	<0.001
CAT, U/mL	60.37 ^b^	41.47 ^a^	56.76 ^b^	2.369	<0.001
SOD, U/mL	348.19 ^b^	262.04 ^a^	272.52 ^a^	11.623	<0.001
NO	210.70 ^b^	149.46 ^a^	190.60 ^b^	8.738	0.001

CON = control group; LPS = LPS challenge group; G-L = GRAM + LPS challenge group; GSH-Px, glutathione peroxidase; CAT, catalase; SOD, superoxide dismutase. Different lowercase letters in the same line indicate significant differences (*p* < 0.05). SEM = standard error of means.

## Data Availability

The original contributions presented in the study are included in the article/Supplementary Material, further inquiries can be directed to the corresponding author.

## Supplementary Materials

The following supporting information can be downloaded at: https://www.mdpi.com/article/10.3390/antiox13050578/s1, Table S1: Analysis composition of basal diets and nutrient level (air-dry basis, %).
