# Supplementary material for: Effects of Crude Extract of Glycyrrhiza Radix and Atractylodes macrocephala on Immune and Antioxidant Capacity of SPF White Leghorn Chickens in an Oxidative Stress Model
Source: Antioxidants (Basel). 2024 May 8;13(5):578. doi: 10.3390/antiox13050578 (PMC11118435; doi:10.3390/antiox13050578)
Supplement: Supplementary file 1 [file antioxidants-13-00578-s001.zip › antioxidants-2966971-supplementary.pdf]

Table S1. Analysis composition of basal diets and nutrient level (air-dry basis, %).

Ingredient (g/kg)	Inclusion
Corn	610.62
Soybean meal, (CP 43%)	336
Limestone, (Ca 38%)	18
Phosphate, (21/27%)	12
Vegetable oil	9
Salt	4
Mineral premix[1)]	2.5
DL-methionine，99%	3.700
L-lysine，50%	3.500
L-threonine	0.640
Total	1000
Nutrient concentrations[2)]	
Dry matter (%)	88.300
Crude protein (%)	21.000
Metabolizable energy (Mcal/kg)	3.087
Choline (mg/kg)	2.000
Arginine (%)	1.210
Linoleic acid (%)	1.200
Total lysine (%)	1.150
Total calcium (%)	1.050
Methionine + cystine (%)	0.840
Valine (%)	0.830
Total Threonine (%)	0.820
Isoleucine (%)	0.790
Total methionine (%)	0.530
Phosphorus available (%)	0.500
Digestible phosphorus (%)	0.480
Total tryptophan (%)	0.210
Total chlorine (%)	0.200
Total sodium (%)	0.200

[1)] The premix provided the following per kilogram diet: vitamin A 10, 000 IU, vitamin D_3 2000 IU, vitamin E 10 IU, vitamin K_3 2.5 mg, vitamin B_1 1 mg, vitamin B_2 6 mg, vitamin B_3 10 mg, vitamin B_5 40 mg, vitamin B_6 3 mg, vitamin B_{11} 0.3 mg, vitamin B_{12} 0.01 mg, biotin 0.12 mg, Cu (as copper sulfate) 8 mg, Fe (as ferrous sulfate) 80 mg, Mn (as manganese sulfate) 60 mg, Zn (as zinc sulfate) 40 mg, Se (as sodium selenite) 0.15 mg, I (as potassium iodide) 0.35 mg.

[2)] Calculated nutrient concentrations.
